# Unfolding the downloads of datasets: A multifaceted exploration of influencing factors

**DOI:** 10.1038/s41597-024-03591-8

**Published:** 2024-07-11

**Authors:** Zhifeng Liu, Pengcheng Luo, Xinglong Tang, Jimin Wang, Lei Nie

**Affiliations:** 1 Department of Information Management, Peking University, Beijing, 100871 China; 2 Peking University Library, Beijing, 100871 China; 3 Country and Area Studies Academy, Beijing Foreign Studies University, Beijing, 100089 China

**Keywords:** Social sciences, Scientific community

## Abstract

Scientific data are essential to advancing scientific knowledge and are increasingly valued as scholarly output. Understanding what drives dataset downloads is crucial for their effective dissemination and reuse. Our study, analysing 55,473 datasets from 69 data repositories, identifies key factors driving dataset downloads, focusing on interpretability, reliability, and accessibility. We find that while lengthy descriptive texts can deter users due to complexity and time requirements, readability boosts a dataset’s appeal. Reliability, evidenced by factors like institutional reputation and citation counts of related papers, also significantly increases a dataset’s attractiveness and usage. Additionally, our research shows that open access to datasets increases their downloads and amplifies the importance of interpretability and reliability. This indicates that easy access enhances the overall attractiveness and usage of datasets in the scholarly community. By emphasizing interpretability, reliability, and accessibility, this study offers a comprehensive framework for future research and guides data management practices toward ensuring clarity, credibility, and open access to maximize the impact of scientific datasets.

## Introduction

Scientific research and production are vital drivers for a country to develop all types of industries. Scientific data are generated from and utilized in both scientific research and production. Scientific data, also called research data^1^, refers to the data produced during the studies and kept for subsequent application by researchers in their studies. Scientific data holds much value in scientific research and production. To consider scientific data disposable may lead to waste due to a massive loss of data value and poor scientific research and production efficiency. Therefore, researchers have begun to reuse scientific data previously shared in their studies to fully utilize the data’s value, improve the efficiency of scientific research and production, and extend the system of comprehensive scientific knowledge. The global recognition and practice of open access have allowed researchers to enhance their understanding of published studies through verification and reanalysis and to inform future investigations^2,3^.

To evaluate the extent of dataset reuse in open access and emphasize the value of releasing datasets, one measure is proposed to assess dataset reuse, which refers to the extent to which a dataset impacts other studies^4,5^. Zeroauli *et al*. (2019) extracted reuse metrics, including dependency, downloads, stars, forks, pull requests, and subscribers^6^. It is meaningful to investigate the factors that may impact the reuse of an open dataset and help improve scholarly communication and scientific productivity. Studies have focused on the analysis of factors affecting the dataset’s reuse. For example, Rolland and Lee^7^ found that the dataset’s structure influences its reuse^7^. Peters *et al*.^8^ noted that the types of datasets have various effects on their citations and altmetrics^8^. The study by Faniel *et al*.^9^ highlights that various data attributes, including completeness, accessibility, ease of operation, and credibility, exhibit positive associations with data reusers’ satisfaction^9^. Silvello^10^ concluded that data attribution, connection, discovery, sharing, impact, and reproducibility have been identified as six primary motivations for data citation^10^. However, most of these previous studies have investigated the effect of one factor or several independent factors on the dataset’s reuse, and composite factors or dimensions that may impact the dataset’s downloads remain to be identified and analysed. Moreover, no previous study has created a conceptual model of the dataset’s downloads to illustrate the specific mechanism of these factors’ influence on the dataset’s downloads. Therefore, there is a need for a study to understand the factors that affect a dataset’s downloads and the specific mechanism of their impact.

Although many studies have discussed and confirmed the significantly positive impact of a dataset’s ease of operation, credibility, and accessibility on its downloads, a systematic analysis of these factors and their belonging dimensions–interpretability, reliability, and accessibility–has not been conducted. Understanding the impact of these dimensions on the dataset’s downloads helps to obtain insights into the nature of the dataset’s downloads. Previous research has suggested that interpretability, reliability, and accessibility may be related to dataset reuse intentions^11,12^. However, the specific mechanism of their influence on the dataset’s downloads remains to be considered, and the interaction mechanisms between different variables are still unclear. To address the research gaps, this study mainly focuses on the three dimensions of factors—interpretability, reliability, and accessibility—to carry out the research and aims to explore the following research questions:

RQ1: How is the interpretability of a dataset associated with its downloads?

RQ2: How is the reliability of a dataset associated with its downloads?

RQ3: How is the accessibility of a dataset associated with its downloads?

RQ4: What are the moderating effects of these factors on the relationships between other factors and downloads?

This study utilizes regression analysis to identify and analyze the research questions. First, it illustrates the theoretical background and develops the hypotheses and conceptual model. Then, it introduces the data and variables used in this research. Subsequently, it presents the analysis and results. Finally, it provides an in-depth discussion of the findings, including their implications, limitations, and directions for future research.

## Methodology

In this section, we present the hypotheses formulated for this study. We then give an in-depth exposition of the data and its pre-processing procedures. Finally, we elucidate the variables as defined in this research.

### Hypothesis development

#### Cognitive load in determining dataset downloads

According to the Unified Theory of Acceptance and Use of Technology (UTAUT) proposed by Venkatesh *et al*.^13^, the actual use of technology is determined by behavioural intention. It reveals four key constructs that directly influence the perceived likelihood of adopting technology: effort expectancy, performance expectancy, social influence, and facilitation conditions. Effort expectancy refers to the degree of ease associated with using technology. In our situation, the dataset’s interpretability is similar to effort expectancy because the ease associated with using the dataset usually depends on the extent to which the dataset can be understood via the attached descriptive text. Unless users thoroughly understand the dataset, they will have trouble using it and may even quit. Therefore, how the dataset can be understood via the attached descriptive text may directly influence and endogenously determine the perceived likelihood of the dataset becoming popular.

More precisely, at the level of cognition, Sweller *et al*.^14^ postulated that the way information is presented (extraneous cognitive load) and the architecture of new information (germane cognitive load) externally determine how well information is grasped^14^. Applied to datasets, if the presented descriptive text attached to a dataset is easy to interpret, it reduces the extraneous cognitive load on the users, making it more likely for the dataset to be used and, thus, to be popularized. Methods to reduce the extraneous cognitive load on the users from the descriptive text for a dataset may strengthen interpretability and make it easier for users to understand. The length of the descriptive text for a dataset is a common metric for measuring the difficulty of understanding scientific expressions. Experiments have suggested that using text that is too long prevents humans from understanding clearly because the brain understands information by grasping and memorizing keywords, and expression with text that is too long increases the cognitive load for the brain’s keyword processing, leading to forgetting the needed information and difficulty in understanding important events for application^15^. Expressions with text that are too long may even lead to human self-protection and the discarding of the content. Thus, the length of the descriptive text for a dataset may negatively affect the dataset’s downloads.

On the other hand, if the descriptive text attached to a dataset that is intended for the users to help understand the architecture is composed of obscure terms, it increases the relevant cognitive load on the users when encountering new information, making users have more trouble in using the dataset and thus damaging the dataset’s downloads; therefore, the readability of the descriptive text is also vital to be considered for the extent of the cognitive load about the descriptive text. The greater the readability of the descriptive text is, the greater the dataset’s downloads are. Here, we propose two hypotheses about the dataset’s interpretability.

**Hypothesis 1a (H1a): The length of the descriptive text for a dataset is negatively correlated with the dataset’s downloads**.

**Hypothesis 1b (H1b): The readability of the descriptive text for a dataset is positively correlated with the dataset’s downloads**.

#### Validated quality in deciding on dataset downloads

UTAUT also reveals that performance expectancy is a core factor for using technology. Performance expectancy refers to the degree to which an individual believes that using technology will help them attain performance gains. In our situation, the dataset’s reliability has a similar meaning to performance expectancy, as users’ perception about the support to scientific research provided by the dataset is strongly associated with the dataset’s assessed value for usage. From the perspective of signalling theory^16^, data producers and users are usually situated in information asymmetry, so data producers can deliver value from the dataset by releasing many relevant signals to make the dataset gain downloads and thus acquire academic reputation. Therefore, when users assess that the dataset is of high value through the shown signals, it confirms that the dataset has value for usage and supports their scientific research; therefore, they choose the dataset for their research, and the dataset’s downloads will be promoted. In this way, data producers and users gradually enter into a “double-win” situation.

The Information Systems Success Model (ISSM) proposed by DeLone and McLean (1992) concludes that the quality of an information system directly impacts users’ assessed value and satisfaction with transforming an information system into an application^17^. Specifically, validated high quality is vital to strengthen the dataset’s reliability and acquire users’ trust. From the perspective of source validation, the authority of the data producer or author is a crucial way to confirm whether the dataset is of high quality. When researchers evaluate the data quality according to the data production process, before reusing it, they must assess the authority of the data producer^18^. In addition, the ranking of the dataset producer’s institution is one of the most typical metrics for measuring the authority of the data producer^19^. The ranking of the dataset author’s institution may positively influence the dataset’s downloads. From the perspective of impact validation, citations of the literature associated with a dataset are caused by the dataset’s reuse. Citations of the literature associated with the dataset largely reflect the literature’s influence and the dataset’s quality in the field, thus further influencing the dataset’s downloads^20^. The number of citations of the literature associated with a dataset may positively correlate with the dataset’s downloads. If a dataset is reliable, it is more likely to satisfy users’ needs and gain their trust in scientific research; therefore, it is more likely to be used, thus increasing its downloads. Here, we propose two hypotheses about the dataset’s reliability.

**Hypothesis 2a (H2a): The ranking of the dataset author’s institution is positively correlated with the dataset’s downloads**.

**Hypothesis 2b (H2b): The number of citations of the literature associated with a dataset is positively correlated with the dataset’s downloads**.

#### Dataset openness in determining dataset downloads and moderating others’ impact

Access to the dataset is a prerequisite for the dataset’s downloads^21^. A dataset having more open files for download can be a direct cause of more download counts and, thus, greater downloads. There may be cases where a dataset is not allowed access. Regardless of its usefulness or ease of use for scientific research and production, the dataset will not be fully downloaded and applied and, thus, will not gain downloads. Additionally, the dataset’s openness moderates the relationship between other endogenous factors and the dataset’s downloads. On the one hand, the openness of data provides an opportunity for users to understand the uploaded dataset and sometimes have a better understanding of the dataset via descriptive visualization^22^. On the other hand, the importance of open data is that it is often associated with increased public trust because open access to datasets with no or few restrictions makes users feel that the data producer has confidence in the uploaded dataset and that the dataset can be used responsibly and that it is trustworthy enough for usage^23^. Here, we propose three hypotheses about the dataset’s accessibility.

**Hypothesis 3a (H3a): The openness of a dataset is positively correlated with the dataset’s downloads**.

**Hypothesis 3b (H3b): The openness of a dataset plays a role as a positive moderator in the relationship between the dataset’s interpretability and downloads**.

**Hypothesis 3c (H3c): The openness of a dataset plays a role as a positive moderator in the relationship between the dataset’s reliability and downloads**.

**Fig. 1:**
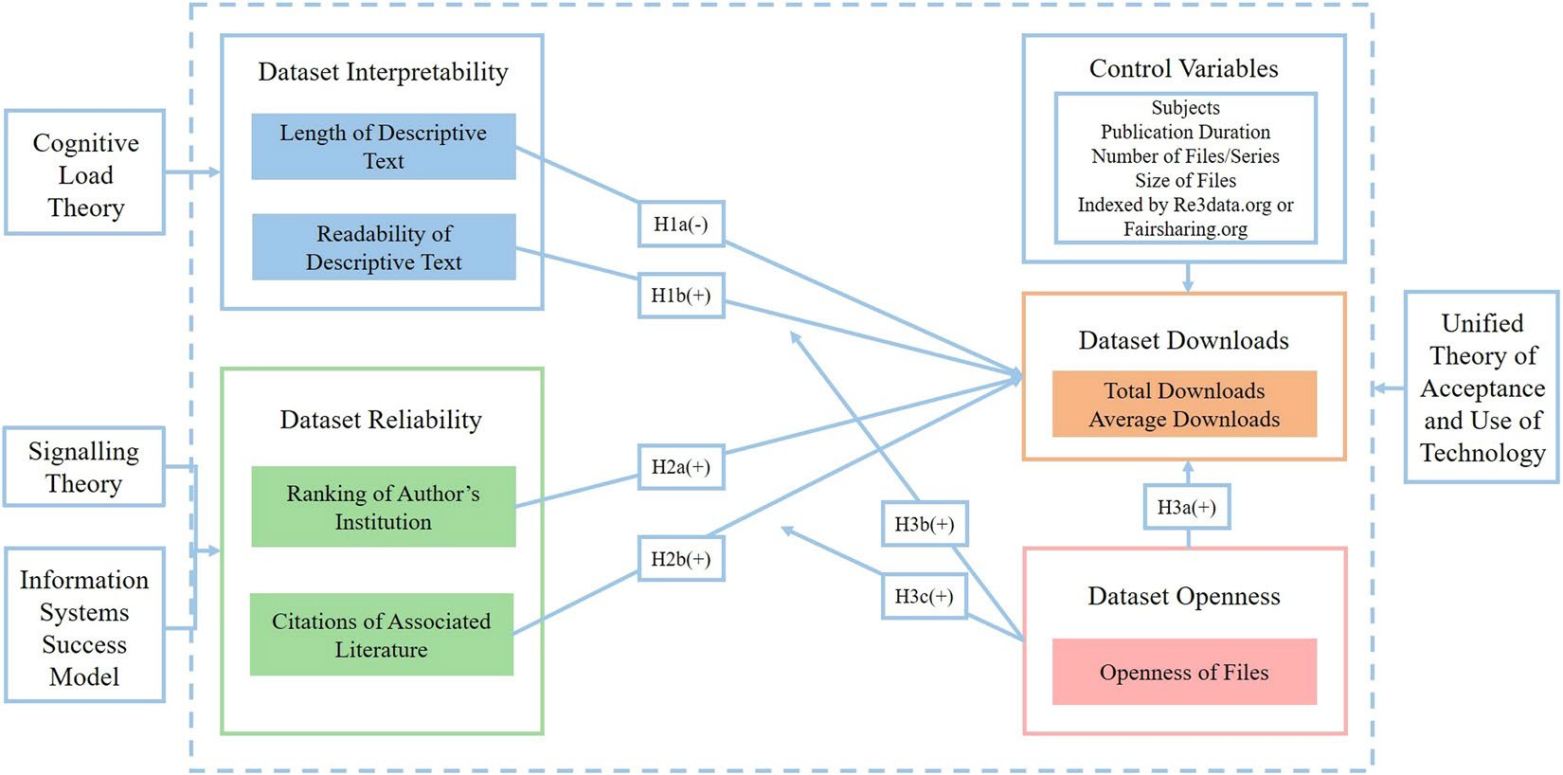
Conceptual diagram of relationships between variables in the research model.

Thus, we finally construct a conceptual model depicted in Fig. 1 to illustrate the focal hypotheses of this study.

### Data

**Fig. 2:**
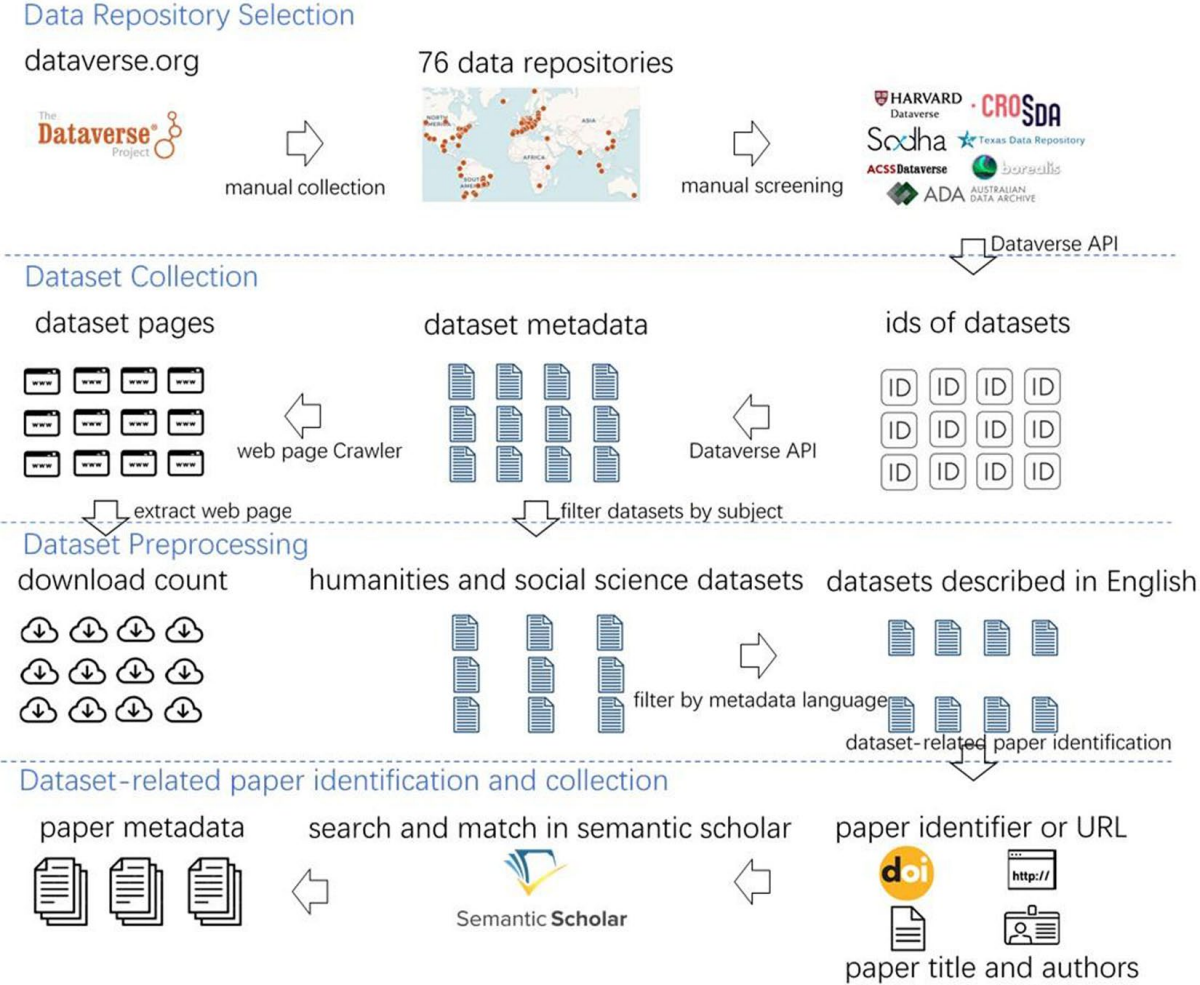
Steps of data collection and pre-processing.

To analyse the factors that influence the downloads of datasets, we selected some representative research data repositories to collect the dataset metadata. We performed pre-processing steps on the collected datasets, such as subject and language identification. We also identified the dataset-related papers and obtained their metadata information from Semantic Scholar. Figure 2 gives the detailed steps of data collection and pre-processing.

### Dataset collection and pre-processing

(1) Research data repository selection and dataset collection

This study systematically examined the datasets in research data repositories by operating on the Dataverse platform. Dataverse, an acclaimed open-source platform for research data management, exerts a significant global impact. As of November 12, 2023, it has been implemented by over 100 institutions worldwide. On February 2nd, 2022, we extracted data on 76 Dataverse-based research data repositories from the dataverse.org website. A portion of these repositories were found to be either devoid of data or inaccessible during our data collection; consequently, this led to the selection of 69 repositories as our definitive sample for this investigation.

We utilized the Dataverse API to extract the dataset metadata, encompassing both the descriptions of the datasets and the specific details of the files contained within each dataset. Initially, we employed the Search API or the “Show Contents of a Dataverse Collection” feature of the Native API to acquire the IDs of all datasets hosted in each repository. Following this, we retrieved comprehensive information about each dataset using the “Get JSON Representation of a Dataset” function from the Native API. Our data collection spanned from February 3rd to February 10th, 2022, and was restricted to datasets natively hosted in each repository. In total, metadata from 201,544 datasets were meticulously gathered for analysis. Ultimately, we extracted the total count of downloads of each dataset from the HTML data acquired through web crawling.

(2) Identifying and selecting humanities and social science datasets

The collected datasets are from the social sciences, humanities, art, science, and engineering disciplines. Many science and engineering datasets have only metadata and no data files, making the downloads useless. For example, the Data INRAe repository contains over 80,000 medical, health, and life science datasets, but most datasets only have metadata. Therefore, we focused on datasets pertinent to the humanities and social sciences in the paper. To refine our datasets, we utilized the subject categorization detailed in the metadata, which encompassed 13 distinct fields: the social sciences, arts and humanities, business and management, law, computer and information science, engineering, mathematical sciences, physics, chemistry, astronomy and astrophysics, earth and environmental sciences, agricultural sciences, and medicine, health and life sciences. By extracting and analysing the subject information from the metadata, we selectively curated datasets specifically related to the social sciences, humanities and arts, business and management, and law. This process culminated in procuring 61,188 datasets relevant to the humanities and social sciences.

(3) Identifying and selecting datasets described in English

The collected data repositories come from different countries and may use different languages to describe the datasets. The language affects analysis metrics such as the length of the description and the readability of the text. To eliminate the influence of language, we only selected datasets described in English for analysis. Specifically, the title, keywords, and abstract of the datasets were extracted from the metadata and concatenated together, and then Langdetect, a tool to identify the language of concatenated text, was used to identify the language of the concatenated text. Among all languages, datasets described in English account for the vast majority. Therefore, we selected the humanities and social science datasets whose metadata were recognized as English as the analysis objects for 55,474 datasets.

### Dataset-related paper identification and collection

Shared datasets mainly support academic research and usually have related papers. The citation count of the related papers was to be used in the subsequent analysis, so we needed to identify the dataset-related papers and collect their metadata information. We first extracted the basic information of the papers, such as title, author, and identifier, from the dataset’s metadata. Then, we used this information to match and retrieve the papers’ metadata information in Semantic Scholar, which includes citation counts.

1. Identifying dataset-related papers
  Three methods were used to identify dataset-related papers: ➀ Determine whether the dataset has related papers based on whether the title of the dataset begins with “Replication Data for”, extract the text after “Replication Data for” as the titles of the related papers, and use the authors of the dataset as the authors of the related papers. ➁ Obtain the identifiers or URLs of the related papers from the “Related Publications” metadata field. ➂ Identify the titles and the authors of the related papers based on the citation text in the “Related Publications” metadata field. Specifically, we randomly sampled 1,000 citation texts and manually annotated the titles and the authors. Then, the conditional random field model, BERT + fully connected layer model, and BERT + CRF model were used for training. Finally, the best model, i.e., BERT + fully connected layer, with an F1 value of 0.9230, was selected to identify the titles and the authors of the related papers for the unlabelled citation text.
2. Collecting metadata of dataset-related papers
  Based on the basic information of the papers extracted in the previous step, we obtained detailed information about the papers by matching them in Semantic Scholar. Specifically, we used the following two methods. For the papers with known titles and authors, we employed the keyword search API provided by Semantic Scholar to retrieve the top 10 search outcomes, using the paper titles as the query. We then calculated the edit distance between the paper titles and the titles from the search results. Moreover, we chose the related papers for the datasets based on the smallest edit distance, provided that this distance was less than one-tenth of the length of the paper titles and that at least one of the listed authors matched the provided authors. For the papers of known identifiers or URLs, we employed the “details about a paper” API of Semantic Scholar to gather their detailed metadata, which included citation counts and other information.
  Across all datasets, there were 40,906 datasets whose titles began with “Replication Data for” or had a “Related Publications” metadata field. In the step of “Identifying dataset-related papers”, we used methods➀, ➁, and ➂ to identify dataset-related papers, and the number of dataset-related papers matched in Semantic Scholar was 10,757, 9,972, and 20,088, respectively. After deduplication, we obtained a total of 26,955 dataset-related papers.

### Variables

Table 1 delineates the definitions and descriptions of the variables. Detailed calculations for each variable will be introduced below.

**Table 1:** Summary of the variables.

	Variable	Abbreviation	Description
Dependent variables	Total downloads	Normalized_download	The total number of downloads of files in the dataset.
	Average downloads	Normalized_average_download	The average daily downloads of files in the dataset.
Independent variables	Length of the descriptive text	Description_length	The number of words in the description metadata field.
	New Dale-Chall score	Dale_chall	The New Dale-Chall readability score of the description text in the dataset metadata.
	Degree of file accessibility	File_openness	The proportion of files in the dataset that are completely open and can be directly downloaded by users.
	Authority of the dataset author’s institution	Institution_rank	The impact of the dataset author’s institution in the world.
	Citations of the dataset-related papers	Related_citation	The total number of citations of papers supported by the dataset.
Control variables	Length of time the dataset was released from	Publication_duration	The number of days between the publication date and the most recent date in the dataset.
	Number of files	File_num	Number of files in the dataset.
	Dataset size	File_size	The total size of the files in the dataset.
	Number of subjects	Subject_num	The number of subjects to which a dataset belongs.
	Indexed by the registry of research data repositories	Re3data_fairsharing	Whether the data repository where the dataset comes from is indexed by re3data.org or fairsharing.org.

### Dependent variables

The dependent variables in this paper include the total downloads and the average number of dataset downloads. The specific measurement methods are outlined as follows:

(1) Total downloads

Total downloads refers to the total number of downloads of all files in the dataset. Since the downloads of datasets vary exponentially and may be 0, we normalize them by adding 1 to the original value, taking the logarithm, and dividing it by the maximum value. Let the original total number of downloads of the dataset *d*_*i*_ be ${{download}}_{i}^{{total}}$; then, the normalized total download score is:

$${normalized}{\rm{\_}}{{download}}_{i}^{{total}}=\frac{{\log }({{download}}_{j}^{{total}}+1)}{{\max }({\log }({{download}}_{j}^{{total}}+1)}$$

(2) Average downloads

Average downloads refers to the average daily downloads of all files in the dataset. The publication time will affect the total number of downloads of the dataset. To eliminate the impact of time, we also use the average number of daily downloads as another dependent variable, i.e.,:

$${{download}}_{i}^{{avg}}=\frac{{{download}}_{i}^{{total}}}{{{day}{\rm{\_}}{count}}_{i}}$$

where ${{day\_count}}_{i}$ is the number of days from the first release date to the date reaching the total number of downloads statistically for dataset *d*_*i*_. The average download score is then normalized in the same way as the total download score, i.e.,:

$${{normalized}{\rm{\_}}{download}}_{i}^{{avg}}=\frac{{\log }({{download}}_{i}^{{avg}}+1)}{{\max }({\log }({{download}}_{j}^{{avg}}+1)}$$

### Independent variables

#### Interpretability of dataset

(1) Length of the descriptive text

The length of the descriptive text refers to the number of words in the description metadata field. To calculate this metric, NLTK is used to tokenize the text, and the number of words in the text is counted after removing punctuation. Since there are a small number of datasets with very long descriptive texts, to eliminate the influence of outliers, we select the maximum value after removing the first 1% for normalization of the length values of the descriptive text. Specifically, let the number of words in the description field of dataset *d*_*i*_’s metadata be ${{len}}_{i}^{{description}}$, and the length of the descriptive text score is:

$${{normalized}{\rm{\_}}{description}{\rm{\_}}{length}}_{i}=\frac{\min ({{len}}_{i}^{{description}},{{\max }}_{1 \% }({{len}}_{j}^{{description}}))}{{{\max }}_{1 \% }({{len}}_{j}^{{description}})}$$

where ${{\max }}_{1 \% }({{len}}_{j}^{{description}})$ is the maximum value after removing the first 1%.

(2) New Dale-Chall score

The New Dale-Chall score is used to measure the readability of the description metadata field of the dataset. The New Dale-Chall^24^ vocabulary contains approximately 3,000 familiar words, and words not in this list are considered complex. Then, the readability of the text is evaluated by calculating the proportion of difficult words and the length of sentences. The higher the proportion of difficult words and the longer the average length of sentences, the more difficult the text is to understand and the higher the New Dale-Chall score. Specifically, for a given text, let ASL be the average length of sentences in the text, and PDW be the proportion of difficult words; then, the New Dale-Chall score is:

$${ndc}=\left\{\begin{array}{lc}{ra}{w}_{{score}}, & {if\; PDW}\le 5 \% \\ {ra}{w}_{{score}}+3.6365, & {if\; PDW} > 5 \% \end{array}\right.$$

$${raw}{\rm{\_}}{score}=0.1579\times {PDW}+0.0496\times {ASL}$$

After obtaining the New Dale-Chall score of the descriptive text of each dataset, the original values are normalized using the max-min normalization method. Suppose that the New Dale-Chall score of dataset *d*_*i*_ is ${{ndc}}_{i}$, and the maximum and minimum values of the New Dale-Chall scores in all datasets are max(ndc) and min(ndc), respectively. The normalized New Dale-Chall score of the dataset *d*_*i*_ is:

$${{normalized}{\rm{\_}}{ndc}}_{i}=1-\frac{{{ndc}}_{i}-{\min }({ndc})}{{\max }({ndc})-\min ({ndc})}$$

The longer the text is, the more accurate the performance of the New Dale-Chall in evaluating text readability is. In this study, we set the New Dale-Chall score to 0 for datasets whose descriptive text lengths are less than or equal to 20.

### Dataset accessibility

(1) Degree of file accessibility

Some datasets allow unrestricted direct download of their data files, whereas others require users to register an account, log into the system, and submit an access request to the dataset owners. In this study, the degree of file accessibility refers to the proportion of the files in the dataset that are entirely open and can be directly downloaded by users without logging into the system, applying for data, and other steps. For a dataset *d*_*i*_, its degree of file accessibility is:

$${{file}{\rm{\_}}{accessibility}}_{i}=\frac{{open}{\rm{\_}}{{file}{\rm{\_}}{count}}_{i}}{{{file}{\rm{\_}}{count}}_{i}}$$

where ${open\_}{{file\_count}}_{i}$ is the number of entirely open files in the dataset and ${{file\_count}}_{i}$ is the total number of files in the dataset. In some cases, the dataset only has metadata but no files, and we set the degree of file accessibility of such dataset to 0.

### Dataset reliability

(1) Authority of the dataset author institution

The authority of the dataset author institution refers to the impact of the author’s institution in the world. We use the Nature Index, US News Best Global Universities Rankings, and QS World University Rankings to measure institutional authority. In the metric calculation, let the ranking range of the Nature Index be $[1,{r}_{\max }]$. If an institution *k* enters the ranking, its ranking value is ${r}_{k}\in [1,{r}_{\max }]$. If *k* does not enter the ranking, its ranking value is ${r}_{\max }+1$. Then, the institutional authority score based on the Nature Index is:

$${{authority}}_{k}^{{ni}}=1-\frac{{r}_{k}-1}{{r}_{{\max }}}$$

Similarly, we can obtain the institutional authority scores ${{authority}}_{k}^{{us}}$ and ${{authority}}_{k}^{{qs}}$ based on the US News Best Global Universities Rankings and QS World University Rankings. Then, the authority score of institution *k* is the average of the three scores, i.e.,:

$${{authority}}_{k}=\frac{1}{3}\times \left. ({{authority}}_{k}^{{us}}+{{authority}}_{k}^{{qs}}+{{authority}}_{k}^{{ni}}\right)$$

A dataset may have multiple authors, and authors may be from different institutions. To obtain the institutional authority for a dataset, the scores of different authors are weighted according to the author’s sequence in the dataset. For the dataset *d*_*i*_, assuming there are *n* authors, the calculation of the authority of the dataset author’s institution is:

$${{institutional}{\rm{\_}}{authority}}_{i}=\mathop{\sum }\limits_{k=1}^{n}{{aw}}_{k}\times {{authority}}_{k}$$

where *aw*_*k*_ is the weight of the *k*^th^ author obtained based on the harmonic counting method^25^., i.e.,:

$${{aw}}_{k}=\frac{\frac{1}{k}}{1+\frac{1}{2}+\ldots +\frac{1}{n}}$$

(2) Citations of dataset-related papers

The citations of dataset-related papers refer to the total number of citations of the papers supported by the dataset. Shared datasets are often used to support some research papers. If the supported research papers are highly cited, it may also affect the downloads of the datasets. Let the collection of the related papers of the dataset *d*_*i*_ be *rp*_*i*_; then, the total number of citations of the related papers is calculated according to the following formula:

$${{citation}{\rm{\_}}{rp}}_{i}^{{total}}=\sum _{p\in {{rp}}_{i}}{citation}(p)$$

where *citation*(*p*) is the number of citations of paper *p* in the related papers. Since the numbers of citations of the related papers vary exponentially and may be 0, we normalize it using the same method as total downloads, i.e.,:

$${{normalized}{\rm{\_}}{citation}{\rm{\_}}{rp}}_{i}^{{total}}=\frac{{\log }({{citation}{\rm{\_}}{rp}}_{i}^{{total}}+1)}{{\max }({\log }({{citation}{\rm{\_}}{rp}}_{j}^{{total}}+1)}$$

For datasets without related papers, the score of this metric is 0.

### Control variables

(1) Publication duration

The publication duration is a control variable since the longer a dataset is published, the higher its download likelihood is. Let the timestamp of the first release of the dataset *d*_*i*_ be *t*_*i*_, the maximum and minimum values of the first release timestamps of all datasets be max(*t*) and min(*t*), respectively, and the max-min normalization method is used to normalize the original timestamp value, i.e.,:

$${{publication}{\rm{\_}}{duration}}_{i}=\frac{{t}_{i}-{\min }(t)}{{\max }(t)-{\min }(t)}$$

(2) Number of files

The number of files in a dataset is also included as a control variable because the more files there are in a dataset, the higher its download likelihood is. Let the number of files in the dataset *d*_*i*_ be ${{file\_count}}_{i}$. Since the numbers of files in different datasets vary exponentially and may be 0, we normalize the original value using the same method as total downloads, i.e.,:

$${{normalized}{\rm{\_}}{file}{\rm{\_}}{count}}_{i}=\frac{{\log }({{file}{\rm{\_}}{count}}_{i}+1)}{{\max }({\log }({{file}{\rm{\_}}{count}}_{j}+1)}$$

(3) Dataset size

The larger the file size in a dataset, the more information it contains and the more likely it is to attract users to download the dataset. Therefore, the dataset size is also included as a control variable. We add the sizes of all files in a dataset to obtain the dataset size. Let the size of the dataset ${d}_{i}$ be ${{dataset\_size}}_{i}$. Since the sizes of different datasets vary exponentially and the number may be 0; we normalize the original value using the same method as total downloads, i.e.,:

$${{normalized}{\rm{\_}}{dataset}{\rm{\_}}{size}}_{i}=\frac{{\log }({{dataset}{\rm{\_}}{count}}_{i}+1)}{{\max }({\log }({{dataset}{\rm{\_}}{count}}_{j}+1)}$$

(4) Number of Subjects

A dataset may be classified into multiple subjects, potentially influencing its downloads. Consequently, this study controls for the number of subjects associated with each dataset. If a dataset is categorized into more than one subject, then *Subject_num* is assigned a value of 1; otherwise, *Subject_num* is set to 0.

(5) Indexed by the registry of research data repositories

Being indexed by the registry of research data repositories refers to whether the data repository where the dataset comes from is indexed by re3data.org or fairsharing.org. re3data.org and fairsharing.org are two essential research data repository indices, and they have indexed many research data repositories worldwide. A data repository is indexed by re3data.org or fairsharing.org; datasets originating from these repositories are more likely to increase their visibility and accessibility, thereby influencing the number of downloads. Consequently, this factor is considered as one of the control variables. In this study, if the data repository where the dataset comes from is indexed by re3data.org or fairsharing.org, then we assign it a score of 1 on this metric; otherwise, it is a score of 0.

## Results

### Descriptive analysis

Table 2 presents the descriptive statistical outcomes for the principal variables under consideration. An inspection of the table reveals a compilation of 55,473 datasets. Notably, the mean values for both *File_openness* and *Re3data_fairsharing* exceed 0.9. This indicates that most datasets are openly accessible for downloading, and most are catalogued within the Re3data and Fairsharing data repositories. Furthermore, *Dale_chall* exhibits the highest standard deviation at 31.463, signalling a substantial variation in the readability of descriptive texts across different datasets. Additionally, we can observe that the datasets were published between 2007 and 2022. The values for all variables are normalized to a uniform scale ranging from 0 to 1 using Min-Max Normalization, a standardization measure critical for ensuring the accuracy of the regression analysis results. Note that the variable *File_size* represents the file size, measured in bytes.

**Table 2:** Descriptive Statistics.

Variable	Obs	Mean	Std. Dev.	Min	Max
Download	55473	533.389	41702.778	0	9534208
Description_length	55473	69.001	102.242	0	5673
Dale_chall	55473	42.884	31.463	6.801	75.582
File_openness	55473	0.936	0.233	0	1
Institution_rank	55473	0.257	0.275	0	0.998
Related_citation	55473	13.422	218.654	0	38174
Publication_duration	55473	844.017	920.628	0	5186
File_num	55473	10.181	120.643	0	23147
File_size	55473	2.798e+08	6.100e+09	0	7.964e+11
Subject_num	55473	0.222	0.416	0	1
Re3data_fairsharing	55473	0.959	0.197	0	1

**Fig. 3:**
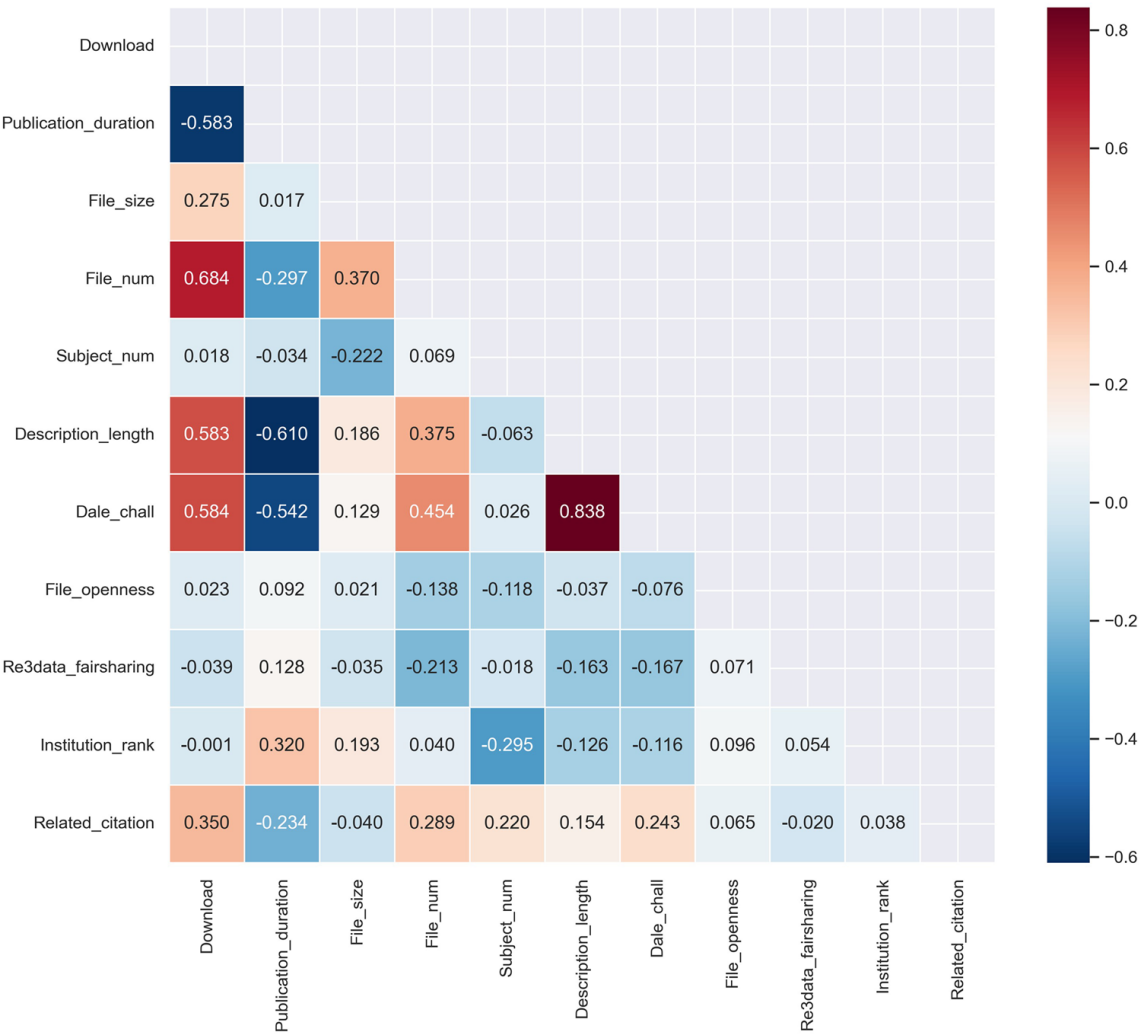
The correlation between the various variables.

Figure 3 delineates the Spearman correlation coefficients among the key variables of interest. Observations from Fig. 3 indicate that the absolute values of the correlation coefficients between most variables are less than 0.5, and all variables have a variance inflation factor (VIF) value below 10, suggesting the absence of multicollinearity among variables. Figure 3 shows that without accounting for other variables, the correlation coefficient between *Dale_chall* and *Download* stands at 0.584, and that between *File_openness* and *Download* stands at 0.023. This denotes a positive correlation between the readability of the dataset description as well as the open accessibility of the dataset files and the downloads of the datasets. Concurrently, the correlation coefficient between *Publication_duration* and *Download* is −0.583, implying that datasets published earlier are more likely to be downloaded and utilized.

### Effects of interpretability and reliability on the downloads of the datasets

The regression results in Table 3 examine the relationships between the interpretability and reliability of the datasets and their subsequent downloads. The baseline model, Model (1), is configured with control variables alone. Extending this foundational model, Model (2) integrates variables that measure the interpretability of the dataset, specifically *Description_length*, and *Dale_chall*.

**Table 3:** OLS regression models of the effects of *interpretability* and *reliability* of dataset on downloads.

Model Variables	Model (1)	Model (2)	Model (3)	Model (4)
Description_length		−0.489^***^		−0.353^***^
		(0.0688)		(0.0571)
Dale_chall		0.0819^***^		0.0705^***^
		(0.00180)		(0.00161)
Institution_rank			0.0670^***^	0.0667^***^
			(0.00197)	(0.00194)
Related_citation			0.277^***^	0.252^***^
			(0.00486)	(0.00474)
Publication_duration	−0.260^***^	−0.181^***^	−0.252^***^	−0.183^***^
	(0.00278)	(0.00346)	(0.00266)	(0.00324)
File_num	0.920^***^	0.842^***^	0.799^***^	0.739^***^
	(0.00871)	(0.00869)	(0.00837)	(0.00833)
File_size	0.0522^***^	0.0525^***^	0.0609^***^	0.0590^***^
	(0.00495)	(0.00481)	(0.00475)	(0.00462)
subject_num	0.00354^***^	−0.00567^***^	0.0122^***^	0.00439^***^
	(0.00119)	(0.00119)	(0.00122)	(0.00122)
Re3data_fairsharing	0.0742^***^	0.0813^***^	0.0706^***^	0.0773^***^
	(0.00281)	(0.00292)	(0.00282)	(0.00288)
_cons	0.125^***^	0.0313^***^	0.0987^***^	0.0178^***^
	(0.00428)	(0.00507)	(0.00404)	(0.00469)
*N*	55473	55473	55473	55473
*R*^2^	0.533	0.569	0.600	0.627
adj. *R*^2^	0.533	0.569	0.600	0.627

Standard errors in parentheses.

*p < 0.1, **p < 0.05, ***p < 0.01.

The regression coefficient associated with *Description_length* is negative, recorded at −0.489, and is statistically significant at the 0.01 level. This outcome indicates an inverse relationship between the dataset’s length of description and downloads, substantiating Hypothesis H1a. Conversely, the coefficient for *Dale_chall* is 0.0819, greater than 0, and is also significant at the 0.01 level, indicating a positive correlation between the readability of dataset description and downloads; therefore, Hypothesis H1b is supported.

Model (3) augments Model (1) by introducing a set of three variables reflective of the reliability of the datasets, which are *Institution_rank*, and *Related_citation*. The coefficients for *Institution_rank* and *Related_citation* are also positive, calculated to be 0.0670 and 0.277, respectively, and both are significantly different from 0 at the 0.01 level. These results affirm a positive link between datasets’ reliability and downloads, thus providing empirical validation for Hypotheses H2a to H2b. Model (4) consolidates variables from both interpretability and reliability aspects, as previously explored in Models (2) and (3). The outcomes of this integrative model sustain the established relationships, demonstrating alignment with the previous results of models.

We conducted three distinct robustness checks to ascertain the robustness of the impact of the interpretability and reliability of the datasets on downloads. Initially, we substituted the dependent variable with the average number of downloads, and the outcomes are shown in Table 4. Subsequently, we excluded samples where the dependent variable was 0, and the regression results of this analysis are displayed in Table 5. Finally, we replaced the OLS regression with a Tobit regression; these results are delineated in Table 6. The consistency across all results reinforces the robustness of our research findings.

**Table 4:** OLS regression models of the effects of *interpretability* and *reliability* of the dataset on the average number of downloads.

Model Variables	Model (1)	Model (2)	Model (3)	Model (4)
Description_length		−0.0241^**^		−0.0111
		(0.0105)		(0.0105)
Dale_chall		0.00221^***^		0.00124^***^
		(0.000368)		(0.000362)
Institution_rank			0.00600^***^	0.00615^***^
			(0.000664)	(0.000691)
Related_citation			0.0230^***^	0.0222^***^
			(0.00195)	(0.00198)
File_num	0.213^***^	0.210^***^	0.202^***^	0.201^***^
	(0.00434)	(0.00455)	(0.00456)	(0.00472)
File_size	0.0121^***^	0.0123^***^	0.0128^***^	0.0128^***^
	(0.00166)	(0.00166)	(0.00169)	(0.00168)
subject_num	0.00180^***^	0.00162^***^	0.00260^***^	0.00253^***^
	(0.000383)	(0.000389)	(0.000391)	(0.000399)
Re3data_fairsharing	0.0210^***^	0.0211^***^	0.0206^***^	0.0207^***^
	(0.000651)	(0.000633)	(0.000642)	(0.000625)
_cons	−0.0433^***^	−0.0439^***^	−0.0450^***^	−0.0454^***^
	(0.00106)	(0.00104)	(0.00105)	(0.00103)
*N*	55473	55473	55473	55473
*R*^2^	0.353	0.353	0.361	0.361
adj. *R*^2^	0.352	0.353	0.361	0.361

Standard errors in parentheses.

*p < 0.1, **p < 0.05, ***p < 0.01.

**Table 5:** OLS regression models of the effects of *interpretability* and *reliability* of the dataset on downloads, excluding samples where the dependent variable was 0.

Model Variables	Model (1)	Model (2)	Model (3)	Model (4)
Description_length		−0.574^***^		−0.425^***^
		(0.0801)		(0.0651)
Dale_chall		0.0223^***^		0.0167^***^
		(0.00226)		(0.00198)
Institution_rank			0.0598^***^	0.0581^***^
			(0.00171)	(0.00173)
Related_citation			0.218^***^	0.216^***^
			(0.00411)	(0.00410)
Publication_duration	−0.0414^***^	−0.0487^***^	−0.0529^***^	−0.0577^***^
	(0.00306)	(0.00342)	(0.00282)	(0.00308)
File_num	0.799^***^	0.797^***^	0.709^***^	0.709^***^
	(0.00807)	(0.00802)	(0.00753)	(0.00750)
File_size	0.0430^***^	0.0444^***^	0.0464^***^	0.0474^***^
	(0.00642)	(0.00643)	(0.00591)	(0.00592)
subject_num	0.00813^***^	0.00890^***^	0.0159^***^	0.0163^***^
	(0.00143)	(0.00143)	(0.00137)	(0.00137)
Re3data_fairsharing	0.0754^***^	0.0709^***^	0.0694^***^	0.0662^***^
	(0.00279)	(0.00291)	(0.00277)	(0.00283)
_cons	0.0264^***^	0.0303^***^	0.0183^***^	0.0209^***^
	(0.00447)	(0.00490)	(0.00413)	(0.00447)
*N*	31191	31191	31191	31191
*R*^2^	0.443	0.450	0.533	0.536
adj. *R*^2^	0.443	0.449	0.533	0.536

Standard errors in parentheses.

*p < 0.1, **p < 0.05, ***p < 0.01.

**Table 6:** Tobit regression models of the effects of *interpretability* and *reliability* of the dataset on downloads.

Model Variables	Model (1)	Model (2)	Model (3)	Model (4)
Description_length		−0.489^***^		−0.353^***^
		(0.0292)		(0.0273)
Dale_chall		0.0819^***^		0.0705^***^
		(0.00123)		(0.00116)
Institution_rank			0.0670^***^	0.0667^***^
			(0.00159)	(0.00153)
Related_citation			0.277^***^	0.252^***^
			(0.00370)	(0.00359)
Publication_duration	−0.260^***^	−0.181^***^	−0.252^***^	−0.183^***^
	(0.00246)	(0.00277)	(0.00240)	(0.00266)
File_num	0.920^***^	0.842^***^	0.799^***^	0.739^***^
	(0.00515)	(0.00509)	(0.00493)	(0.00487)
File_size	0.0522^***^	0.0525^***^	0.0609^***^	0.0590^***^
	(0.00422)	(0.00406)	(0.00392)	(0.00379)
subject_num	0.00354^***^	−0.00567^***^	0.0122^***^	0.00439^***^
	(0.00107)	(0.00104)	(0.00102)	(0.000997)
Re3data_fairsharing	0.0742^***^	0.0813^***^	0.0706^***^	0.0773^***^
	(0.00222)	(0.00215)	(0.00206)	(0.00201)
_cons	0.125^***^	0.0313^***^	0.0987^***^	0.0178^***^
	(0.00347)	(0.00376)	(0.00328)	(0.00354)
*N*	55473	55473	55473	55473

Standard errors in parentheses.

*p < 0.1, **p < 0.05, ***p < 0.01.

### Effects of accessibility on the downloads of the datasets

Table 7 illustrates the regression analysis results examining the impact of accessibility on dataset downloads. In Model (1) within Table 7, the variable *File_openness*, representing the dataset’s accessibility, is included along with its interaction terms with *Description_length*, *Dale_chall*, *Institution_rank*, and *Related_citation*. The coefficient for *File_openness* is 0.072, which is statistically significant at the 0.01 level. This indicates that open access to datasets can enhance their downloads, validating Hypothesis H3a.

**Table 7:** OLS regression models of the effects of dataset accessibility on downloads.

Model Variables	Model (1)	Model (2)	Model (3)
Description_length	−0.242^***^	−0.415^***^	−0.242^***^
	(0.0553)	(0.0751)	(0.0284)
Dale_chall	0.0695^***^	0.0155^***^	0.0695^***^
	(0.00158)	(0.00212)	(0.00115)
Institution_rank	0.0712^***^	0.0546^***^	0.0712^***^
	(0.00189)	(0.00179)	(0.00151)
Related_citation	0.234^***^	0.204^***^	0.234^***^
	(0.00461)	(0.00412)	(0.00354)
File_openness	0.0720^***^	0.0503^***^	0.0720^***^
	(0.00229)	(0.00339)	(0.00177)
File_openness * Description_length	−0.370^***^	−0.757^***^	−0.370^***^
	(0.104)	(0.130)	(0.0757)
File_openness * Dale_chall	0.0552^***^	0.0391^***^	0.0552^***^
	(0.00534)	(0.00733)	(0.00413)
File_openness * Institution_rank	0.121^***^	0.0877^***^	0.121^***^
	(0.00732)	(0.0114)	(0.00535)
File_openness * Related_citation	0.175^***^	0.225^***^	0.175^***^
	(0.0225)	(0.0274)	(0.0164)
Publication_duration	−0.186^***^	−0.0605^***^	−0.186^***^
	(0.00315)	(0.00308)	(0.00262)
File_num	0.761^***^	0.716^***^	0.761^***^
	(0.00812)	(0.00747)	(0.00485)
File_size	0.0261^***^	0.0444^***^	0.0261^***^
	(0.00485)	(0.00596)	(0.00382)
subject_num	0.00721^***^	0.0183^***^	0.00721^***^
	(0.00120)	(0.00137)	(0.000981)
Re3data_fairsharing	0.0718^***^	0.0615^***^	0.0718^***^
	(0.00290)	(0.00288)	(0.00197)
_cons	0.167^***^	0.115^***^	0.167^***^
	(0.00342)	(0.00360)	(0.00281)
*N*	55473	31191	55473
*R*^2^	0.643	0.545	
adj. *R*^2^	0.643	0.544	

Standard errors in parentheses.

*p < 0.1, **p < 0.05, ***p < 0.01.

The interaction term between *File_openness* and *Description_length* is negative and statistically significant, suggesting that open access can strengthen the relationship between the dataset’s length of description and downloads. Then, the interaction term between *File_openness* and *Dale_chall* is positive and significant at the 0.01 level, suggesting that open access enhances the relationship between the readability of dataset description and downloads; Hypothesis H3b is confirmed.

Additionally, the coefficients for the interaction terms between *File_openness* and the two variables denoting dataset reliability, *Institution_rank*, and *Related_citation*, are all positively significant. These results suggest that open access to datasets notably reinforces the positive correlation between their reliability and downloads, substantiating Hypothesis H3c.

To verify the robustness of our findings, we conducted two robustness tests. The first entailed the exclusion of samples where the dependent variable was 0, followed by a regression analysis utilizing the revised sample set, the results of which are encapsulated in Model (2), presented in Table 7. The second test involved transitioning from an OLS regression framework to a Tobit regression analysis, with the corresponding results detailed in Model (3) of Table 7. Notably, the outcomes of both Model (2) and Model (3) are in alignment with the results of Model (1), thereby indicating a high degree of robustness in the findings.

## Discussion

### Main findings

Scientific data is the fundamental basis for research and scientific progress. At the same time, the amount of scientific data available is increasing rapidly, resulting in a new form of scholarly output. This study explores how the interpretability, reliability, and accessibility of scientific datasets affect their usage and impact, with the goal of improving open sharing and reusability of scientific data.

Regarding the interpretability of the datasets, our findings reveal a contrasting influence of descriptive text attributes on dataset downloads. Studies show that datasets with longer descriptions receive fewer downloads. This suggests that overly lengthy descriptions may deter users, possibly due to the increased complexity or time required to understand the dataset. In contrast, the readability of the descriptive text positively affects dataset downloads, indicating that user engagement is likely to be higher with datasets that present their information in a more comprehensible and understandable manner. Staron and Scandariato argue that data with low interpretability is treated equivalently to data with low veracity^26^. Low veracity data may result in a decline in dataset downloads. This study highlights the importance of balancing comprehensive information with clarity and conciseness in dataset description, as the length of description and readability have contrasting effects on the audience.

Moreover, this research indicates that the reliability of the datasets, as evidenced by the reputation of the producing institution, and the volume of the related academic citations, significantly boosts their downloads. Previous research has also indicated that there are concerns regarding dataset reliability that must be addressed before researchers can confidently utilize data collected by others^7^. These findings underscore the pivotal role of perceived reliability and scholarly recognition in enhancing a dataset’s appeal and usage within the academic community, highlighting the value of establishing and maintaining credible and visible data sources.

Additionally, our study reveals that the openness of dataset files directly enhances dataset downloads and amplifies the impact of both interpretability and reliability on downloads. Similarly, previous scholars have found that, after accounting for other factors affecting citation rates, there is a robust citation benefit associated with open data, a finding that aligns with the results of our research^27^. This finding underscores the synergistic effect of open access to datasets, which not only fosters immediate user engagement but also reinforces the positive impacts of clear, readable dataset descriptions and reliable data sources. Thus, open access creates a compound effect in increasing a dataset’s attractiveness and usage within the scholarly community.

### Theoretical contributions and implications

Previous studies have employed qualitative research methods to explore how and why scholars utilize scientific data from repositories. Some researchers have used survey methods and identified that the data producer and data quality are significantly related to researchers’ trust and data reuse^28^. Other studies, adopting the scholars’ perspective, have investigated data reuse behaviors, revealing that perceived efficacy, efficiency, and the importance of data reuse are critical factors influencing scholars’ use and reuse of scientific data^11^. In contrast to these studies, the present research focuses on the impact of data interpretability, reliability, and accessibility on scholars’ data downloading and reuse behaviors. This study makes several theoretical contributions.

First, this study applies the Unified Theory of Acceptance and Use of Technology (UTAUT) to the context of dataset downloads, drawing an analogy between effort expectancy in the UTAUT framework and the dataset’s interpretability. The dataset’s interpretability is akin to effort expectancy because the ease of using the dataset generally hinges on how comprehensible the dataset is through its accompanying descriptive text. Additionally, by integrating this theory with cognitive load theory, the research reveals the nuanced balance between the extent and clarity of the dataset description, emphasizing the importance of readability rather than merely the length of the description. This approach enriches the theoretical connotations of both the UTAUT and cognitive load theory.

Second, this study also applies the performance expectancy component of the UTAUT framework to analyze the impact of dataset reliability on users’ downloads. Adopting and downloading a dataset can be compared to adopting a new technology. The reliability of the dataset is just as important as the expected performance, as users’ perception of the dataset’s value for scientific research is strongly linked to its perceived usefulness. Furthermore, this study incorporates signalling theory, utilizing the authority of the dataset’s authors and their institutions as indicators of dataset reliability. Finally, by integrating with the Information Systems Success Model (ISSM), the study confirms a positive correlation between the authority of the dataset’s authors and their institutions and the dataset’s downloads, innovatively linking UTAUT and signalling theory. This novel approach provides theoretical value for subsequent research.

Third, this study not only uncovers a positive relationship between dataset openness and dataset downloads, addressing the previous analytical focus limited to dataset citations but also reveals the positive moderating effect of dataset openness on the relationships among dataset interpretability, reliability, and downloads. This further underscores the importance of dataset openness. Overall, the research constructs and validates a comprehensive conceptual framework of factors influencing dataset downloads, thereby contributing significantly to the existing literature.

### Implications for practice

From a practical perspective, this research offers actionable guidance for data curators, librarians, and policy-makers in scientific data management. First, it is crucial to balance comprehensiveness and conciseness in dataset descriptions. Data managers should ensure that descriptions are clear and concise, avoiding overly lengthy narratives that may deter potential users. Data managers can enhance the quality of these descriptions by setting prompts on data platforms that encourage data owners to use succinct and clear language and implement a word limit to keep descriptions focused and to the point. Additionally, providing examples of effective descriptions from highly downloaded datasets as references could serve as a practical guide for data providers.

Second, in order to increase the credibility of an institution’s datasets, it is important to build the institution’s reputation and encourage academic citations. One way to achieve this is by establishing partnerships with academic institutions to promote research that utilizes the datasets. This will enhance the visibility and perceived reliability of the datasets. Additionally, repositories should conduct regular audits on the quality of their datasets to ensure their integrity and reliability. By implementing this quality control process, the datasets become more trustworthy and appealing to researchers.

Third, the findings emphasize the importance of having unrestricted access to datasets. To make accessing data easier without any unnecessary restrictions, repositories should adopt and promote open data policies. This can be achieved by revising licensing agreements to make them more flexible and providing adequate infrastructure for easy data downloading and processing. Additionally, repositories should actively encourage data owners to open their data files and promote open datasets through various communication channels. While advocating for open data policies, it is essential to acknowledge and address the complexities and sensitivities that may arise. For instance, considerations regarding data sensitivity, privacy, and ethical concerns must be meticulously evaluated.

### Limitations and future research

This research constructs an impact factor model for dataset downloads, encompassing interpretability, reliability, and accessibility, which enriches our understanding of the mechanism behind dataset reuse and dissemination. However, certain limitations in this study warrant further exploration.

First, the current analysis predominantly focuses on internal factors influencing dataset downloads. This narrow focus may neglect the broader context in which data is shared and utilized, including the technological platforms that also play significant roles in the accessibility and utility of datasets. Future studies will extend this scope by examining external factors, such as the influence of data platforms.

Second, this study has examined the relationship between the interpretability, reliability, and accessibility of datasets and dataset downloads. Future research could further explore the relationships between the interpretability, reliability, and accessibility of datasets and other metrics, such as the number of users of the datasets and the citations of the datasets.

Third, this study exclusively utilizes the ranking of the author’s institution and citations of associated literature to represent dataset reliability and investigates the relationship between dataset reliability and downloads. The aspect of dataset trust, a significant element of dataset reliability, was not considered due to the absence of effective measures. Future research could propose new metrics for dataset trustworthiness and explore the relationship between dataset trustworthiness and downloads. Besides, the primary analysis in this study is based on social science dataset samples. This choice limits the scope of findings to a specific scientific domain, which may reduce the applicability of the conclusions across different fields that may have unique data characteristics and user communities. Future research will benefit from incorporating natural science datasets to diversify and expand the analytical samples, thereby broadening the study’s applicability and enhancing the generalizability of its findings.

Lastly, this study employs a quantitative approach, analyzing the factors influencing scholars’ downloading and usage of datasets based on 55,473 datasets from 69 data repositories through regression analysis. This method may not fully reveal the underlying intentions behind scholars’ downloading and usage behaviors. Therefore, subsequent research will incorporate qualitative methods, including surveys and interviews, to investigate how and why scholars utilize scientific data from repositories. This combined approach aims to provide deeper insights into scholars’ data usage patterns, ultimately promoting the reuse of scientific data.

## Data Availability

The data examined in this research is available in the Figshare repository (10.6084/m9.figshare.24721209.v3)^29^.

## Code availability

The code used in this research is available in the Figshare repository (10.6084/m9.figshare.24721209.v3)^29^.
